# Optimizing the strain engineering process for industrial-scale production of bio-based molecules

**DOI:** 10.1093/jimb/kuad025

**Published:** 2023-09-01

**Authors:** Eric Abbate, Jennifer Andrion, Amanda Apel, Matthew Biggs, Julie Chaves, Kristi Cheung, Anthony Ciesla, Alia Clark-ElSayed, Michael Clay, Riarose Contridas, Richard Fox, Glenn Hein, Dan Held, Andrew Horwitz, Stefan Jenkins, Karolina Kalbarczyk, Nandini Krishnamurthy, Mona Mirsiaghi, Katherine Noon, Mike Rowe, Tyson Shepherd, Katia Tarasava, Theodore M Tarasow, Drew Thacker, Gladys Villa, Krishna Yerramsetty

**Affiliations:** Inscripta, Inc., 5720 Stoneridge Dr, Suite 300, Pleasanton, CA 94588, USA; Inscripta, Inc., 5720 Stoneridge Dr, Suite 300, Pleasanton, CA 94588, USA; Inscripta, Inc., 5720 Stoneridge Dr, Suite 300, Pleasanton, CA 94588, USA; Inscripta, Inc., 5720 Stoneridge Dr, Suite 300, Pleasanton, CA 94588, USA; Inscripta, Inc., 5720 Stoneridge Dr, Suite 300, Pleasanton, CA 94588, USA; Inscripta, Inc., 5720 Stoneridge Dr, Suite 300, Pleasanton, CA 94588, USA; Inscripta, Inc., 5720 Stoneridge Dr, Suite 300, Pleasanton, CA 94588, USA; Inscripta, Inc., 5720 Stoneridge Dr, Suite 300, Pleasanton, CA 94588, USA; Inscripta, Inc., 5720 Stoneridge Dr, Suite 300, Pleasanton, CA 94588, USA; Inscripta, Inc., 5720 Stoneridge Dr, Suite 300, Pleasanton, CA 94588, USA; Inscripta, Inc., 5720 Stoneridge Dr, Suite 300, Pleasanton, CA 94588, USA; Inscripta, Inc., 5720 Stoneridge Dr, Suite 300, Pleasanton, CA 94588, USA; Inscripta, Inc., 5720 Stoneridge Dr, Suite 300, Pleasanton, CA 94588, USA; Inscripta, Inc., 5720 Stoneridge Dr, Suite 300, Pleasanton, CA 94588, USA; Inscripta, Inc., 5720 Stoneridge Dr, Suite 300, Pleasanton, CA 94588, USA; Inscripta, Inc., 5720 Stoneridge Dr, Suite 300, Pleasanton, CA 94588, USA; Inscripta, Inc., 5720 Stoneridge Dr, Suite 300, Pleasanton, CA 94588, USA; Inscripta, Inc., 5720 Stoneridge Dr, Suite 300, Pleasanton, CA 94588, USA; Inscripta, Inc., 5720 Stoneridge Dr, Suite 300, Pleasanton, CA 94588, USA; Inscripta, Inc., 5720 Stoneridge Dr, Suite 300, Pleasanton, CA 94588, USA; Inscripta, Inc., 5720 Stoneridge Dr, Suite 300, Pleasanton, CA 94588, USA; Inscripta, Inc., 5720 Stoneridge Dr, Suite 300, Pleasanton, CA 94588, USA; Inscripta, Inc., 5720 Stoneridge Dr, Suite 300, Pleasanton, CA 94588, USA; Inscripta, Inc., 5720 Stoneridge Dr, Suite 300, Pleasanton, CA 94588, USA; Inscripta, Inc., 5720 Stoneridge Dr, Suite 300, Pleasanton, CA 94588, USA; Inscripta, Inc., 5720 Stoneridge Dr, Suite 300, Pleasanton, CA 94588, USA

**Keywords:** Biomanufacturing, Genome editing, Genome engineering, Industrial strain engineering, Synthetic biology

## Abstract

Biomanufacturing could contribute as much as ${\$}$30 trillion to the global economy by 2030. However, the success of the growing bioeconomy depends on our ability to manufacture high-performing strains in a time- and cost-effective manner. The Design–Build–Test–Learn (DBTL) framework has proven to be an effective strain engineering approach. Significant improvements have been made in genome engineering, genotyping, and phenotyping throughput over the last couple of decades that have greatly accelerated the DBTL cycles. However, to achieve a radical reduction in strain development time and cost, we need to look at the strain engineering process through a lens of optimizing the whole cycle, as opposed to simply increasing throughput at each stage. We propose an approach that integrates all 4 stages of the DBTL cycle and takes advantage of the advances in computational design, high-throughput genome engineering, and phenotyping methods, as well as machine learning tools for making predictions about strain scale-up performance. In this perspective, we discuss the challenges of industrial strain engineering, outline the best approaches to overcoming these challenges, and showcase examples of successful strain engineering projects for production of heterologous proteins, amino acids, and small molecules, as well as improving tolerance, fitness, and de-risking the scale-up of industrial strains.

## Graphical Abstract

**Graphical Abstract:**
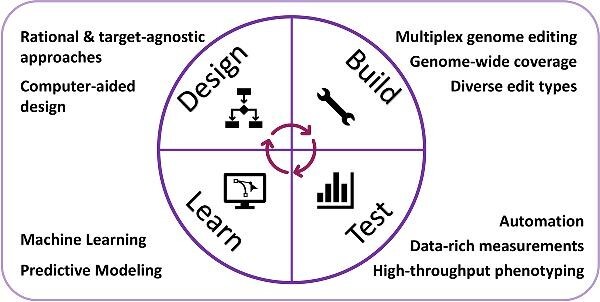
This perspective will discuss methods to overcome the challenges in strain engineering necessary to support the increasing role that biotechnology will play as a disruptive force for several industries.

## Introduction

**Fig. 1.**
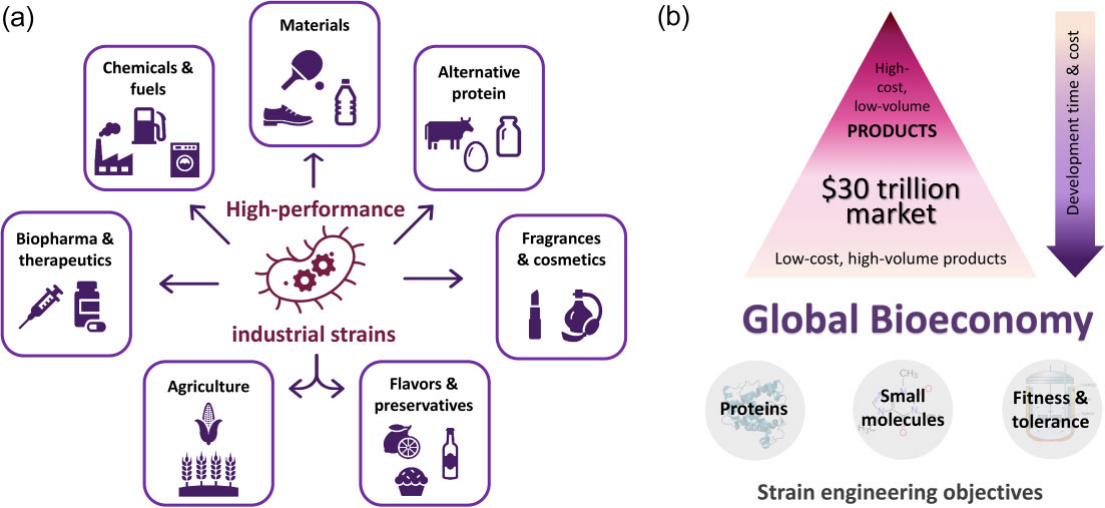
(a) Applications of strain engineering. (b) The size of the potential market for products made using biomanufacturing and strain engineering objectives for reaching it.

Biotechnology is poised to bring disruptive innovation across many industries, from energy to healthcare. The size of the industry is estimated to reach ${\$}$30 trillion by 2030 and could account for more than a third of the global output of manufacturing industries (Candelon et al., 2022). Microbial biomanufacturing of chemicals, materials, and biomolecules promises to reduce greenhouse gas emissions, improve land use, and bolster sustainability by replacing the products derived from petroleum and ingredients sourced from animals or wild-harvested plants with sustainable alternatives made from renewable feedstocks (Fig. 1a) (Scown & Keasling, 2022). However, doing so requires developing efficient and robust industrial strains capable of producing a wide range of products, from small molecules to proteins, at competitive prices. Currently, the majority of commercialized bio-based products are high-value molecules, such as active pharmaceutical ingredients (APIs), flavors and fragrances, and specialty chemicals supporting relatively small markets. At the same time, most of the opportunity remains untapped primarily due to the inability of biomanufacturing routes to compete with the price points established by the existing production methods. Reaching those profit margins requires extreme strain performance, which is difficult to achieve with traditional strain development approaches (Fig. 1b). In order to capture the market opportunities across all sectors of the bioeconomy, we need to improve the efficiency of the strain engineering process to reduce development costs and time to market.

Our ability to engineer biological systems has improved dramatically thanks to technological advances in genome engineering and interrogation tools over the last several decades. DNA sequencing, synthesis, and assembly technologies, CRISPR (clustered regularly interspaced short palindromic repeats) genome editing, and other synthetic biology tools have greatly accelerated and elevated the strain engineering process. However, alongside the progress in manipulating genomes came the full appreciation that biology is incredibly complex and not as easy to engineer as man-made modular systems. A landmark publication titled “Five hard truths for synthetic biology” details the obstacles that stand in the way of fully utilizing synthetic biology to predictably engineer biological systems (Kwok, 2010). Among those hard truths are the complexity and unpredictability of biological systems, the fact that many of the genetic parts are incompatible or undefined, and that introducing a high degree of variability can render biological systems inoperable. This sentiment has been repeatedly corroborated by industry and academic research that has demonstrated the difficulty of transferring established systems to different chassis (Wang et al., 2011) and the diminished fitness resulting from strain “over-engineering” (Colletti et al., 2011; Van Dien, 2013).

The takeaway from these studies is that our ability to predictably engineer organisms to achieve specific phenotypic outcomes remains limited, and that there is no universal approach to successful strain engineering. Instead, to effectively engineer optimized production strains, we must use both rational and empirical approaches and develop novel, high-dimensional data sets to assess how well strains perform in manufacturing conditions. Decreasing the cost of strain engineering and reducing process development timelines are essential for bringing commercially competitive bio-based products to market. An important driver will be integrating these technologies with data-driven approaches to be able to better predict which strains will perform best in large-scale manufacturing conditions. This comprehensive approach to strain development will be the focus of this perspective.

## The Framework for Effective Strain Engineering

The Design–Build–Test–Learn (DBTL) cycle framework is a widely used iterative process of strain development that is carried out until the desired performance is achieved. The Design and Build stages encompass the process of genetic manipulation of cells, where Design broadly represents the strategy and Build represents the tools and techniques employed for physically introducing sequence diversity. Test encompasses phenotyping methods and workflows, whereas Learn refers to computational tools used to analyze the data collected during testing. The Test and Learn stages can be conceptually grouped as procedures for phenotyping, connecting the genotype to the associated phenotype, and drawing conclusions or making predictions about which genetic changes generate the desired strain improvement goals. The Learn stage subsequently informs the Design phase of the next DBTL cycle and is instrumental in improving our ability to build better strains with each engineering cycle. To reduce strain development timelines and costs, each of the cycles needs to be optimized and their number decreased. In the following sections, we describe the advances in conceptual understanding of how we can optimize each of the four phases and demonstrate how successful integration of the entire DBTL cycle can produce significant improvements in the turnaround times and cost of strain engineering projects.

### Design

**Fig. 2.**
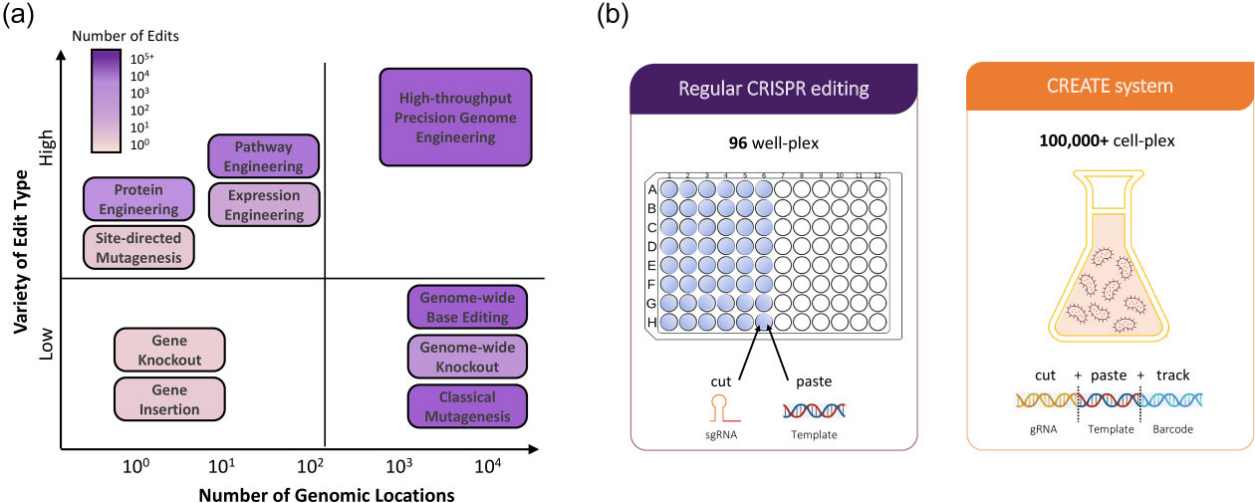
(a) Methods for genome editing, their throughput, and genome access capabilities (Fox, 2019). (b) The CREATE method enables building large (up to 10 000 variants in a single experiment) and diverse libraries with edits of varying length, and allows tracking of the edited cell populations via high-throughput sequencing of the barcodes located on the editing plasmids (Garst et al., 2017). See text for further details.

Design strategies for strain improvement span a spectrum of approaches for generating genetic diversity: from rational (e.g., integration of defined and specific edits) to semi-rational (e.g., enzyme variants and hundreds to thousands of hypothesis-driven targets) to completely random (e.g., through chemical mutagenesis). These approaches are not mutually exclusive but often complementary, and their selection is informed by the phenotyping capacity and confidence of the hypotheses being tested (Fig. 2a). Rational design implementation has been highly successful in many cases, including microbial production of artemisinin (Ro, 2006) or 1,4-butanediol (1,4-BDO) (Yim et al., 2011), or improving polyketide production by increasing cellular malonyl-coenzyme A concentration (Zha et al., 2009). In addition, there are exciting new developments in the use of artificial intelligence (AI) methods for protein designs, which could improve enzyme performance within biochemical pathways. For example, large language models have been used to successfully design proteins and predict amino acid substitutions that are more likely to preserve folding and function and therefore increase discovery rates by enriching for candidates that are more likely to be functional (Hesslow et al., 2022; Madani et al., 2023). However, rational engineering strategies are often insufficient for achieving desired strain improvement goals when used alone, which is why random approaches such as mutagenesis, adaptive laboratory evolution (ALE), and directed evolution methods are still widely used for improving productivity and robustness of industrial strains. Rational and target-agnostic approaches complement each other and must be used together to achieve necessary strain performance.

Random or target-agnostic strain engineering approaches are often used to solve difficult design problems. For example, ALE is often used to obtain complex phenotypes such as tolerance and fitness but typically requires a long time for a population to evolve the desired trait over many generations. ALE can be accelerated by using media supplemented with a chemical mutagen (Mundhada et al., 2016), UV exposure (Alcántara-Díaz et al., 2004), enhanced recombination (Winkler & Kao, 2012; Peabody et al., 2016), or by deleting genes required for mismatch repair (Kang et al., 2019). In one example, separate *Escherichia coli* populations were evolved in the presence of 11 different inhibitory compounds, which resulted in 89 populations that adapted to tolerate concentrations 60%–400% higher than initial toxic levels (Lennen et al., 2023). Sequencing genomes of 223 isolates from those 89 populations, followed by reverse engineering of recurring mutations, helped uncover some of the tolerance mechanisms, including mutations in the transcription factor subunit *rpoA* (discussed in more detail below).

A drawback of ALE experiments is that it generates a limited spectrum of mutations (typically, single nucleotide polymorphisms or large deletions) and requires tedious and expensive follow-up experiments to identify and isolate the causal mutations. Mutational burden, also known as Muller's ratchet (Andersson & Hughes, 1996), may introduce negative downstream effects on fitness and potentially mask or reduce the effect of the causal mutations (Couce et al., 2017). For these reasons, follow-up studies are necessary to confirm and reconstitute each mutation in a clean genetic background before moving forward to the next cycle of strain engineering. Another significant downside of random mutagenesis is that it leaves many potentially beneficial mutations inaccessible. Mutations in a gene are much more likely to cause a loss than a gain in function, as supported by bacterial and yeast evolution studies (Cooper et al., 2001; Kvitek & Sherlock, 2013; Venkataram et al., 2016). Additionally, gain-of-function mutations often result from amino acid changes that require more than one nucleotide change within the codon (Pines et al., 2017, 2022). Statistically speaking, the probability of two random mutations occurring in the same codon is very low, necessitating the use of targeted gene editing methods. For these reasons, while random approaches are essential, the status quo for decades leaves much to be desired. The ideal approach would combine the specificity and versatility of rational design with the breadth of random approaches.

### Build

Comprehensive and effective strain engineering typically requires multiple build tools to introduce diverse edit types and access targets across the entire genome. The emergence of CRISPR-based editing has greatly facilitated genome exploration for enhanced function discovery. However, there are still tradeoffs between throughput and cost, being able to make precise edits, variety of edit types (deletions, insertions, or substitutions), the size of the edit (number of nucleotides), and the number of genomic locations accessible to editing (single locus vs. genome wide) (Fig. 2a). For example, classical methods, such as chemical, UV, or transposon mutagenesis (Alcántara-Díaz et al., 2004; van Opijnen et al., 2009; Mundhada et al., 2016), and global transcription machinery engineering (Alper et al., 2006), are easy to implement and can access the whole genome, but the changes generated are completely random and require extensive deconvolution experiments to understand. On the other hand, precise edits such as saturation mutagenesis, recombineering, and CRISPR-based editing require significant effort, time, and expertise to execute (Ronda et al., 2015; Stovicek et al., 2015; Roy et al., 2018; Lian et al., 2019). Additionally, an important limitation of the Build phase is the need to balance the number of edits with the screening capacity of the Test phase.

Precision editing across the genome has been historically associated with a one-hypothesis-per-well approach. The CRISPR EnAbled Trackable genome Engineering (CREATE) strategy overcomes this limitation thanks to its ability to incorporate edits throughout the genome in a highly multiplex, trackable manner (Garst et al., 2017). In contrast to traditional well-based precision editing approaches, each cell receives a precise genomic edit introduced via CRISPR/Cas9 recombineering using a plasmid that contains the specified guide ribonucleic acid (RNA) encoding sequence, a donor template sequence encoding the desired edit along with a protospacer adjacent motif (PAM)-deactivating silent (synonymous) mutation, and a short molecular barcode all expressed together on a single cassette (Fig. 2b). This method has demonstrated high editing efficiency and enables tracking of the edited populations by deep sequencing of the barcodes located on the editing plasmid. Thus, CREATE libraries can be used for selection studies where cell populations can be tracked through time or under varied conditions. The CREATE method enables building large and diverse libraries, with edit types that span deletions, insertions, and substitutions, can target regions anywhere in the genome, and is amenable to automation. The high quality and frequency of edits facilitate the Test phase by reducing the number of unedited variants that compete for the phenotyping capacity. In addition, diverse, high-quality libraries improve the success of subsequent engineering cycles by providing rich edit data for the Learn and follow-up Design phases. Overall, this technique overcomes many of the existing technological limitations of genome editing.

### Test

**Fig. 3.**
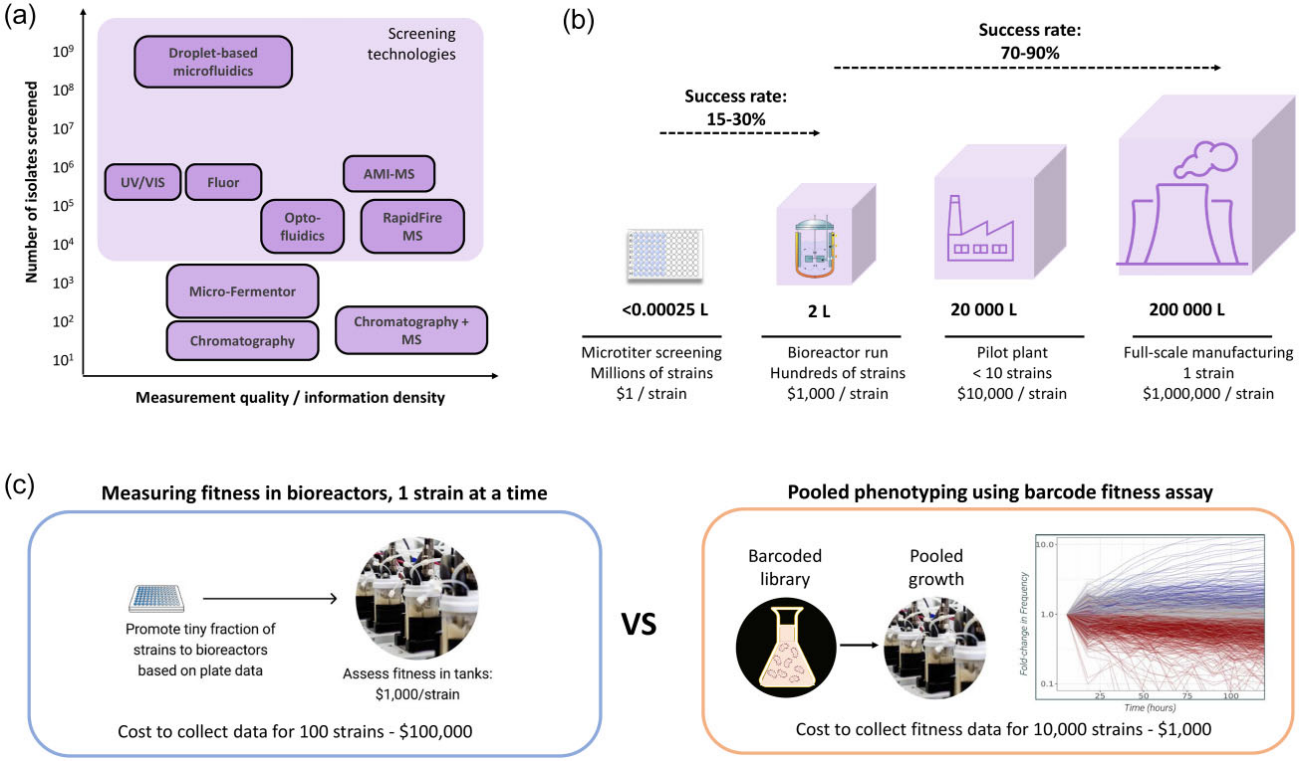
(a) Typical number of isolates screened for different phenotyping methods. These methods are covered in detail elsewhere (Leavell et al., 2020). (b) The “valley of death” is commonly thought to lie in scale-up, but predictive power is weakest when scaling from microtiter to bench fermentation scales. (c) Assessing strain performance in bioreactors can be done much more efficiently and cost-effectively in a pooled format using barcoded strain libraries.

Phenotyping is a critical part of the DBTL cycle that requires specific expertise in designing assays, selection studies, or high-throughput screening methods. Testing large, complex libraries often involves balancing resources with optimizing the quality of data output that feeds into the Learn stage of the cycle. The challenge at this stage is to ensure that the screening capacity of a given strategy matches the size of the edited libraries (Fig. 3a) while simultaneously collecting high-quality, rich data that can be used to predict the success of downstream scale-up efforts and inform subsequent Design efforts. Most testing platforms focus almost entirely on microtiter plate assays yielding limited types and depths of measurements. However, the performance of strains at different cultivation scales can vary significantly, especially between the microtiter scale used for high-throughput screening and fed-batch bioreactors required for strain validation (250 mL–10 L) and manufacturing (up to hundreds of thousands of liters), resulting in the low predictive power of the most relevant performance.

Predicting the performance of strains under manufacturing conditions using standard high-throughput screening data is particularly difficult. Based on collective experience, the observed correlation between microtiter plate assays and the first bioreactor run for mature projects is as low as 15%–30%, in contrast to the much higher predictive power of extrapolating the success rate of scaling from the bioreactor to pilot plant and commercial manufacturing (20 000 –200 000 L) (Fig. 3b). This means that the majority of strains selected for validation based on microtiter plate measurements fail to achieve the expected performance in bioreactors, resulting in significant increases in the time and cost to market. A major contributor to this miscorrelation is the difficulty of assessing strain fitness in plate-based assays. On the other hand, measuring fitness in bioreactor conditions for individual strains is costly, at a cost of several thousands of dollars per bioreactor run. This conflict can be resolved by screening thousands of strains in a single bioreactor in parallel, an approach that is enabled by barcoded library techniques such as CREATE (Fig. 3c).

**Fig. 4.**
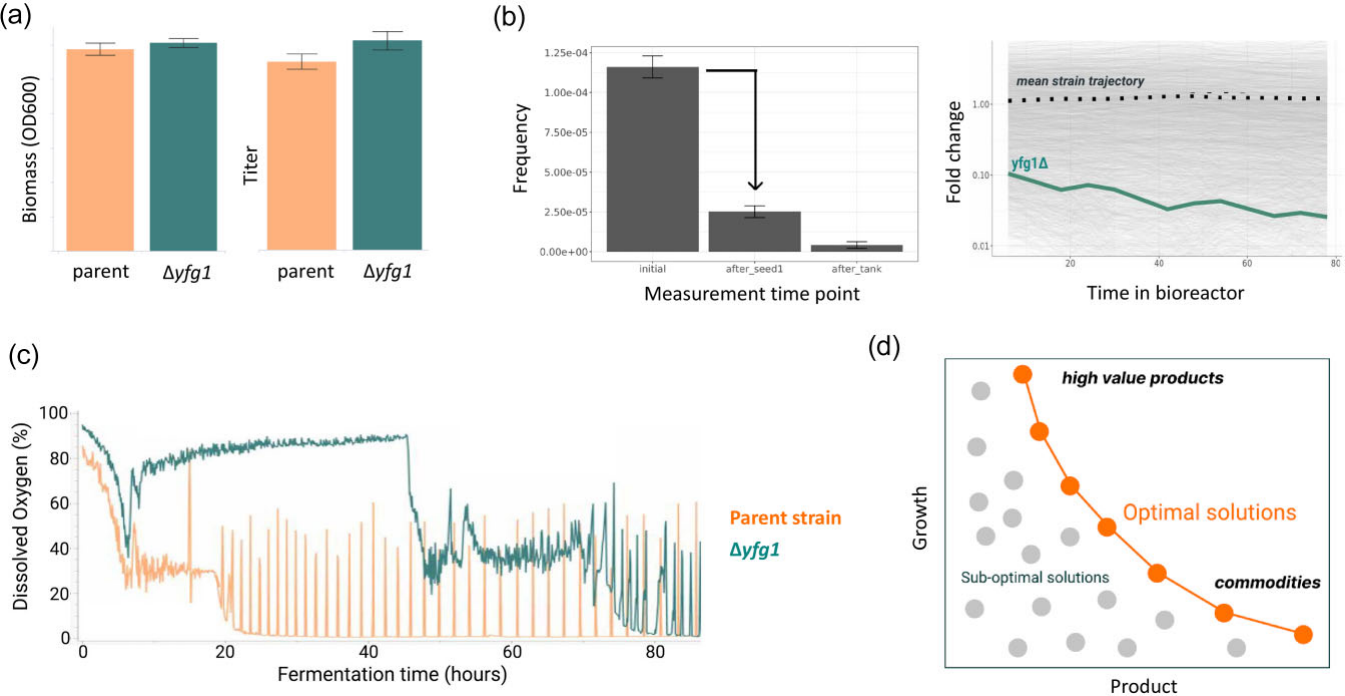
(a) Performance data for a promising scale-up candidate measured in 96-well plate-based assays. (b) Fitness assay predicted reduced the performance of the identified candidate at the bioreactor scale. (c) Bioreactor run data showed that the strain performed much worse than the parent strain, as predicted by the fitness score. (d) A theoretical limit between strain growth and product titer suggests that developing optimized strains requires intelligent tradeoffs between production and growth. There are likely inadmissible solutions in the commodity regime requiring unrealistically high product titer and low growth as some nominal growth is required for production at all.

As an example of how fitness assessment studies can help de-risk scale-up, one of our internal projects identified a promising variant containing a gene deletion edit based on literature reports. This variant showed both high titer and biomass yield in microtiter plate assays, suggesting it would be a good candidate for scale-up (Fig. 4a). We conducted a pooled bioreactor run to assess the fitness of edited strains, including this edit, in parallel. During the fitness assay, barcode abundance was measured throughout the run and used to calculate fitness scores. The barcode associated with the candidate strain showed a strong drop in frequency over the course of the bioreactor run, suggesting that this edit had a strong negative effect on fitness in bioreactor conditions (Fig. 4b). To verify this hypothesis, the strain was cultured singly in a bioreactor and compared to the performance of the parent strain (Fig. 4c). As predicted from the bioreactor selection experiment, the edited strain failed to perform in the fed-batch process, as indicated by the dissolved oxygen trace, underscoring the importance of developing predictive measures such as fitness scores to de-risk scale-up efforts. In general, pooled fitness assays can be used to assess entire library populations and quantify the “fitness costs” associated with each beneficial titer gain, with the goal of operating as close to the optimal solution space as possible (Fig. 4d).

Pooled selection studies are similarly useful for engineering complex phenotypes like tolerance or sensitivity. Selection experiments can be designed to select for growth rate, biomass yield, nutrient utilization, tolerance to specific compounds, resistance to antibiotics, or production of a metabolite (Schmidt,^ 2005^; Feist et al., 2010; Lennen et al., 2023). Selection studies must be carefully designed to provide adequate selection pressure and ensure that genomic changes that provide small advantages are not overlooked, as those changes can generate synergistic effects when combined in subsequent cycles of genome engineering. Sequencing of adapted populations provides rich data sets that can capture population dynamics and genotype–phenotype correlations, especially when done at sufficient sequencing depth. Selection studies are facilitated by tools like CREATE, which enables cost-effective sequencing of very large populations by capturing edit data from plasmid-based barcodes as opposed to the depth required for whole-genome sequencing. The availability of high-quality, multidimensional phenotyping and genotyping data increases the success of subsequent DBTL cycles.

### Learn

The Learn phase must take advantage of the vast troves of genotype and phenotype data gathered in the Test phase. Increasingly, machine learning tools play an important role in extracting meaningful data points and making inferences about how to design better editing libraries with each engineering cycle (Fox et al., 2007; Eraslan et al., 2019; Thean et al., 2022; Mathis et al., 2023). As discussed in the Design section above, new AI methods for protein design are rapidly advancing (Hesslow et al., 2022; Madani et al., 2023). In addition to their use in *a priori* design, these tools can also generate models that can be trained on high-throughput phenotyping data sets to further refine genotype–phenotype associations, leading to better designs in the next round of optimization. Overall, the Learn phase can inform subsequent Design cycles, supplement and enhance existing metabolic models, and increase our understanding of the fundamental principles of biology. Some of these principles, or lessons learned, over the last few decades of high-throughput strain engineering using the DBTL framework are outlined below:

**Lesson #1:** Fitness is paramount for manufacturing success. High mutational load weakens the strain and the introduction of genetic modifications designed to confer useful properties often results in slowed growth or other reductions in fitness (Jin & Cate, 2017). However, natural evolutionary adaptations can restore the desired growth phenotype. In one example, an *E. coli* strain recoded to free up the amber stop codon (Lajoie et al., 2013) was propagated for 1000 generations to restore its growth rate, while deep-sequencing of the evolved population revealed the causes of reduced growth (Wannier et al., 2018).

**Lesson #2:** Progress in strain engineering is generally sigmoidal. In other words, it is harder to engineer strains that are already optimized. The fitter the strain, the smaller the effect of introducing additional beneficial mutations. This phenomenon is sometimes referred to as “diminishing returns” epistasis, which has been observed in the experimental evolution of *Methylorubrum extorquens* and *Saccharomyces cerevisiae* (Kryazhimskiy et al., 2014), as well as from long-term evolution studies in *E. coli* (Wang et al., 2016). As the titer approaches the theoretical maximum, the growth rate approaches zero, so optimization efforts must consider balancing growth and fitness along with product titer and yield (Fig. 4d).

**Lesson #3**: Epistasis can generate significant improvements from stacking mutations, but this effect is rarely achieved rationally. Epistatic effects are unpredictable and can obscure phenotypes. These effects are thought to be the underlying mechanism of why the fitness landscape appears to be “rugged” (Yang et al., 2022).

**Lesson #4**: Rugged fitness landscapes are characterized by many local maxima and minima with multiple potential peaks (Weinreich et al., 2005; de Visser & Krug, 2014). To avoid getting stuck in a “fitness valley”, design strategies need to maintain enough diversity when moving into the next cycles of strain engineering. An effective strategy for maintaining genetic diversity is shallow screening of the libraries and recombining advantageous edits to tap into the power of beneficial epistatic effects (Fox & Giver, 2010; Alvizo et al., 2014). Biological design space is essentially infinite, however, and machine learning tools integrated into the DBTL cycle can prove very powerful in constraining the vast combinatorial space to increase the efficiency of the search.

The most important lesson, perhaps, is that to improve the success rate of strain engineering, we must effectively integrate all four steps and utilize the rich data generated in each cycle to inform subsequent engineering efforts. For instance, building high-quality libraries can greatly simplify subsequent testing of those libraries and learning from them. Gathering multiple data points during the Test phase can significantly improve the predictive power of models used for optimizing the performance of strains under different conditions such as during scale-up and aid in the selection of design targets for iterative genome engineering. Ultimately, these efforts are aimed at engineering strains in a more predictable manner and with a higher degree of success. In the next section, we will demonstrate how this approach can facilitate strain engineering for the production of small molecules, amino acids, proteins, and the engineering of complex traits like tolerance and fitness. These represent real-world examples of strain engineering projects undertaken by Inscripta and showcase internally generated (unpublished) data.

## Strain Engineering Examples Using the Integrated DBTL Approach

### Improving Production of Recombinant Proteins

Common industrial microorganisms like *S. cerevisiae* and *E. coli* are used to produce recombinant proteins for a multitude of applications and markets such as skincare, household products, animal feed, textiles, food and beverage, pharmaceutical, chemical, starch and paper processing, bioremediation, biosensor, and waste management industries (Wang et al., 2017; Mital et al., 2021). Typically, strain engineering for recombinant protein production involves improving protein expression by introducing extra copies of the protein-encoding gene into the genome, engineering the promoter and expression sequences of the protein expression cassette, optimizing the protein-encoding gene's codon usage, and improving protein folding and secretion through signal sequence engineering. In addition to improving the expression of the target protein itself, recombinant protein production often requires making additional changes to the host's metabolism to improve amino acid utilization, regulating the stress response, improving protein export from the cell, knocking out or down-regulation of protease or glycosylation genes (for secreted proteins), and other modifications aimed at improving the productivity and fitness of the strain. Development of a production-ready strain can take many months and requires genome-wide approaches to achieve performance targets.

**Fig. 5.**
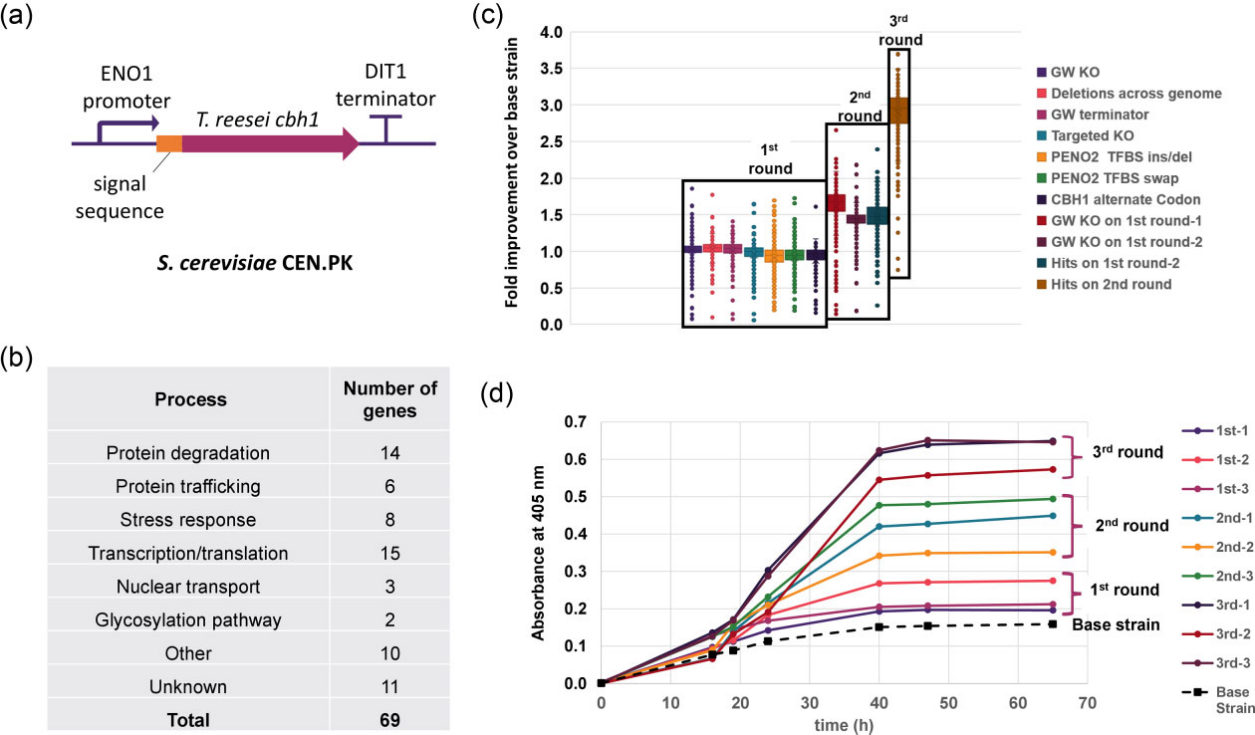
Genome engineering for improved protein production and secretion of heterologous CBH1 protein in *S. cerevisiae*. (a) Schematic of the base strain construct for heterologous CBH1 expression. (b) Summary of top hits identified in the first round of editing and screening and their functional class. (c) Fold improvement in secreted CBH1 activity in screened variants from the first, second, and third rounds of editing. Clustered box plot shows individual variant measurements, which are grouped by library type. (d) Comparison of strain performance of top variants from the first, second, and third rounds of screening in shake flasks. Cultures were grown for 72 hr in YPD medium supplemented with 20 g/L glucose.

Multiplex genome engineering that combines rational and target-agnostic diversity generation strategies can significantly speed up this process. An example that demonstrates the success of this approach is the engineering of *S. cerevisiae* CEN.PK strain for secretion of a cellobiohydrolase I (CBH1) enzyme. Cellobiohydrolases are enzymes involved in cellulose degradation, which are being actively explored for use in cellulosic biomass and consolidated bioprocessing applications. Fungal CBH1 enzymes, such as from *Taloromyces emersonii*, have been successfully expressed in *S. cerevisiae* but at relatively low secretion titers (Den Haan et al., 2007; Ilmén et al., 2011a). To improve the recombinant production of CBH1 in *S. cerevisiae*, a single copy of the *T. emersonii cbh1* gene was introduced into the CEN.PK strain downstream of the LEU2 locus to construct the base strain. The expression cassette contained a native pENO2 constitutive promoter, a native *T. emersonii* secretion signal sequence, and a DIT1 terminator (Fig. 5a). The base strain was then edited using multiplex CRISPR-based editing libraries that introduce stable genomic modifications and can be tracked using a barcode located on the editing plasmid.

The design strategy for the genome-wide editing libraries involved a mix of rational, semi-rational, and target-agnostic approaches. The rational and semi-rational targets were identified based on literature reports and included knockouts (KOs) of genes previously shown to improve heterologous protein production in yeast, KOs of known glycosylation and protein degradation genes to improve heterologous protein stability, ENO2 promoter diversification through insertion, deletion, or swapping of transcription factor binding sites (TFBSs), and a codon optimization library to improve the expression of CBH1 (Penttilä et al., 1988; Ilmén et al., 2011b; Tripathi & Shrivastava, 2019). The genome-wide libraries included untargeted genome-wide knockouts (GW KOs), synthetic terminator insertion library for every coding sequence (CDS), and random genome-wide deletions introduced as a genome-diversification strategy. Following the construction of the edited strain libraries (∼15 000 total edits), 8400 random variants were screened individually for improved CBH1 secretion, as measured using an activity assay with chromophoric substrate pNP-beta-lactopyranoside with a characteristic absorbance at 405 nm.

The first round of screening identified 69 unique hits that fell into diverse functional categories, such as protein degradation, secretion, glycosylation, stress response, transcription and translation, nuclear transport, as well as ENO2 promoter and alternate-codon variants (Fig. 5b). The diverse set of target types contributing to this complex phenotype would be difficult to rationally predict or discover without the ability to systematically interrogate the entire genome. The top 2 variants identified in the first round of screening were then used as base strains for the next editing cycle. In the design of the second round of edits, two different strategies were undertaken: (1) the top hit strain identified in the first round of screening was combined with a 5133-edit genome-wide knockout library; and (2) two top performing strains from the first round of screening were combined with the remaining hits identified in the first screening round. After the second round of editing, the top hit strains identified were again combined with the library of hits from the first screening round. The total time required to complete three rounds of editing, including the library design, preparation, and screening stages, was 6 months.

The results showed a stepwise improvement in secreted CBH1 activity after each round of genome editing (Fig. 5c). In the single-edit libraries, a maximum ∼1.8-fold increase over baseline activity was observed; with two-edits libraries, the activity in screened isolates ranged between 1.6- and 2.3-fold above baseline; the triple-edit strains showed up to a 3.5-fold improvement in microtiter plates. Since the screening was performed in a high-throughput format with isolates grown and assayed in deep-well plates, we wanted to verify that the improvement seen in the engineered strains would translate to a larger scale. The top three isolates from each round were propagated for 72 hr in 25-mL flasks in yeast extract peptone dextrose (YPD) medium supplemented with 20 g/L glucose. The performance in shake flasks matched the performance in microtiter plates, with the top variants demonstrating nearly a 4-fold improvement over base strain (Fig. 5d).

### Engineering Tolerance and Fitness in Industrial Strains

Tolerance to inhibitory compounds is an important trait for industrial strains. Many commercially valuable product classes have inhibitory characteristics or involve the production of toxic intermediates that can slow down the growth rate and reduce the productivity of the strain. One such compound is *p*-coumaric acid (pCA), a phenolic acid found in edible plants that exhibits beneficial health properties, including the ability to decrease low-density lipoprotein peroxidation, antioxidant, and antimicrobial activities (Boz, 2015). However, its concentration in plant sources is low, making extraction difficult and expensive. This presents an opportunity for commercial production in engineered microorganisms. Microbial strains have been engineered to produce pCA at concentrations ranging from 2.3 to 1740 mg/L (Vargas-Tah & Gosset, 2015), but higher titers are limited by product toxicity. pCA demonstrates strong growth inhibition and completely arrests growth in *E. coli* at a concentration of 10 g/L (Sariaslani, 2007; Rodríguez-Ochoa et al., 2022). To overcome this limitation, efforts have been undertaken to adapt strains to higher pCA concentrations using ALE and study the effects of pCA on gene expression (Mohamed et al., 2020; Rodríguez-Ochoa et al., 2022). Both approaches require subsequent studies to discern and validate the mutations responsible for the tolerance phenotype from non-causal changes in the genome and transcriptome.

**Fig. 6.**
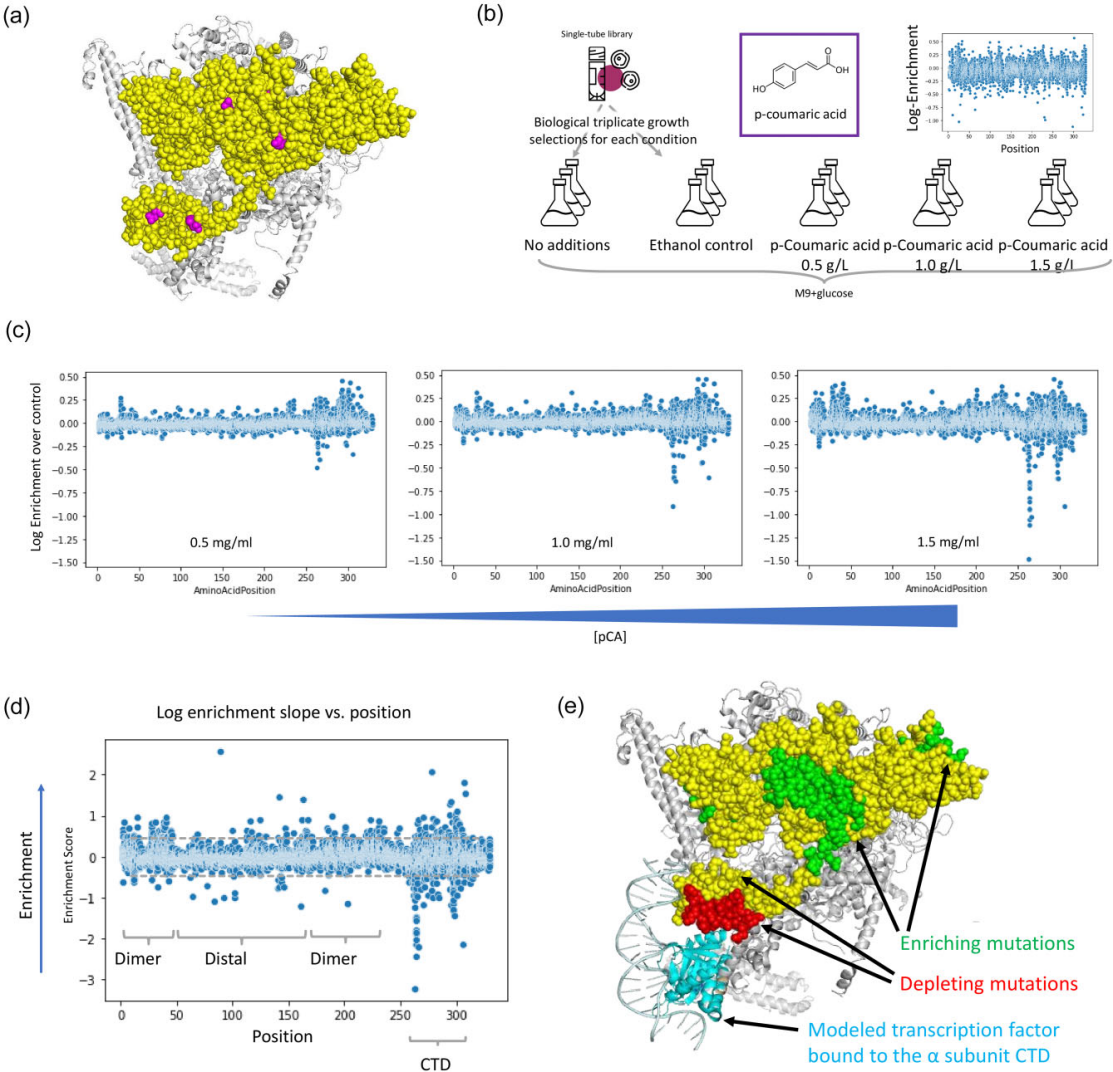
(a) ALE variants discovered by chemical selections previously (Lennen et al., 2023) shown mapped onto the structure of the RNA polymerase PDB 4JK1. The α subunit is colored in yellow with discovered variants colored in magenta. (b) Experimental setup for the p-coumaric acid (pCA) selection study. Enrichment in minimal media over rich media is shown. (c) Deep sequencing of the *rpoA* edit library enabled the identification of edits enriched or depleted in the presence of pCA. (c) Specific edits enriched or depleted in the presence of pCA plotted according to their abundance. (d) Mapping the slope of the log response against concentration yields an enrichment score to identify behaviors of individual mutations. (e) The *rpoA* tolerance and sensitivity mutations determined in this work mapped on the RNA polymerase structure PDB 4JK1 (Mechold et al., 2013) with a transcription factor modeled bound to the CTD (Murakami, 2015 and references therein).

In order to improve tolerance in a more direct manner, while avoiding hitchhiker mutations, we applied CREATE-based engineering to perform saturation mutagenesis of the native on-genome *rpoA* in *E. coli*, which encodes the α-subunit of RNA polymerase​. This subunit plays a key role in the complete assembly of the RNA polymerase through homodimerization in the N-terminal domain and transcription factor binding and regulation in the C-terminal domain (CTD). This protein has been shown to play a role in tolerance to terpenes, ethanol, butanol, organic acids, and phenolic compounds (Klein-Marcuschamer et al., 2009; Minty et al., 2011; Niu et al., 2019; Lennen et al., 2023). Those ALE variants in *rpoA* were captured in a library of growth-inhibiting chemicals (Fig. 6a) (Lennen et al., 2023). A library constructed on *E. coli* MG1655 containing 6880 total single amino acid mutations of *rpoA* was then tested for pCA tolerance​ in a series of concentrations, ranging from 0.5 to 1.5 g/L (Fig. 6b). The plasmid-based barcodes specific to this editing method present the ability to track populations and identify which edited variants are enriched or depleted under increasing concentrations of pCA, essentially testing all 6880 hypotheses simultaneously.

Sequencing of library populations by barcode and on genome grown in the presence of pCA pointed to a wide variety of mutations that give tolerance or sensitivity to the compound (Fig. 6c). Tracking the log enrichment of the population over the behavior of the same variant in the no-pCA control through increasing concentrations of pCA provides an enrichment scoring function associated with the slope, and thus yields variants that are increasing or decreasing in tolerance dependent on concentration (Fig. 6d). Mapping the top 10% of variants for enriching (green) or depleting (red) onto the structure of the α-subunit of RNA polymerase shows clear clustering within specific three-dimensional regions (Fig. 6e). Enriching mutations were clustered primarily in the homodimerization boundary of the complex, as well as being distributed to distal regions on the N-terminus and one surface of the CTD. However, sensitivity-enhancing mutations were alternatively strongly clustered to the CTD specifically, and more interestingly were strongly mapped to the proposed surface of the interaction of transcription factors.

The *rpoA* role as a global regulator is consistent with the wide range of transcriptional responses associated with tolerance. Other studies on the effect of pCA on cellular response have identified induction of genes involved in pCA active export, cell wall, membrane component, and amino acid synthesis, detoxification of formaldehyde, phosphate limitation, acid stress, protein folding, and degradation, as well as downregulation of genes involved in energy production, carbohydrate metabolism, and plasma membrane proteins (Rodríguez-Ochoa et al., 2022). Here we were able to identify sensitivity- and tolerance-associated mutations in a single-genome engineering experiment and pinpoint the specific residues on *rpoA* associated with tolerance. This study demonstrates the versatility of CREATE-based genome editing, showcases how it enables engineering of complex phenotypes like tolerance, and highlights how it facilitates elucidation of causal relationships between genotype and phenotype by way of large and rich experimental data sets.

### Strain Engineering for Production of Amino Acids

Amino acids are used as animal feed as well as in the pharmaceutical, nutraceutical, and other industries, comprising a multibillion-dollar market (Félix et al., 2019). Microbial fermentation is a dominant method for industrial production of l-amino acids such as l-lysine, which is typically produced in engineered *Corynebacterium glutamicum* and *E. coli* (Liu et al., 2022). However, strain engineering for amino acid production is not a trivial task: Endogenous lysine biosynthesis pathways are highly evolved with extensive regulatory features, and thus require genome-wide engineering. Sequencing of a lysine-overproducing *C. glutamicum* strain obtained through decades of adaptive evolution revealed more than 1000 mutations (Yang & Yang, 2017), including beneficial mutations at the single-gene, pathway, and whole-genome levels. To demonstrate the advantages of a genome-wide engineering approach that combines rational and target-agnostic strategies, we selected a wild-type *E. coli* K-12 strain MG1655, which produces a nominal amount (0.01–0.08 mg/L) of lysine.

**Fig. 7.**
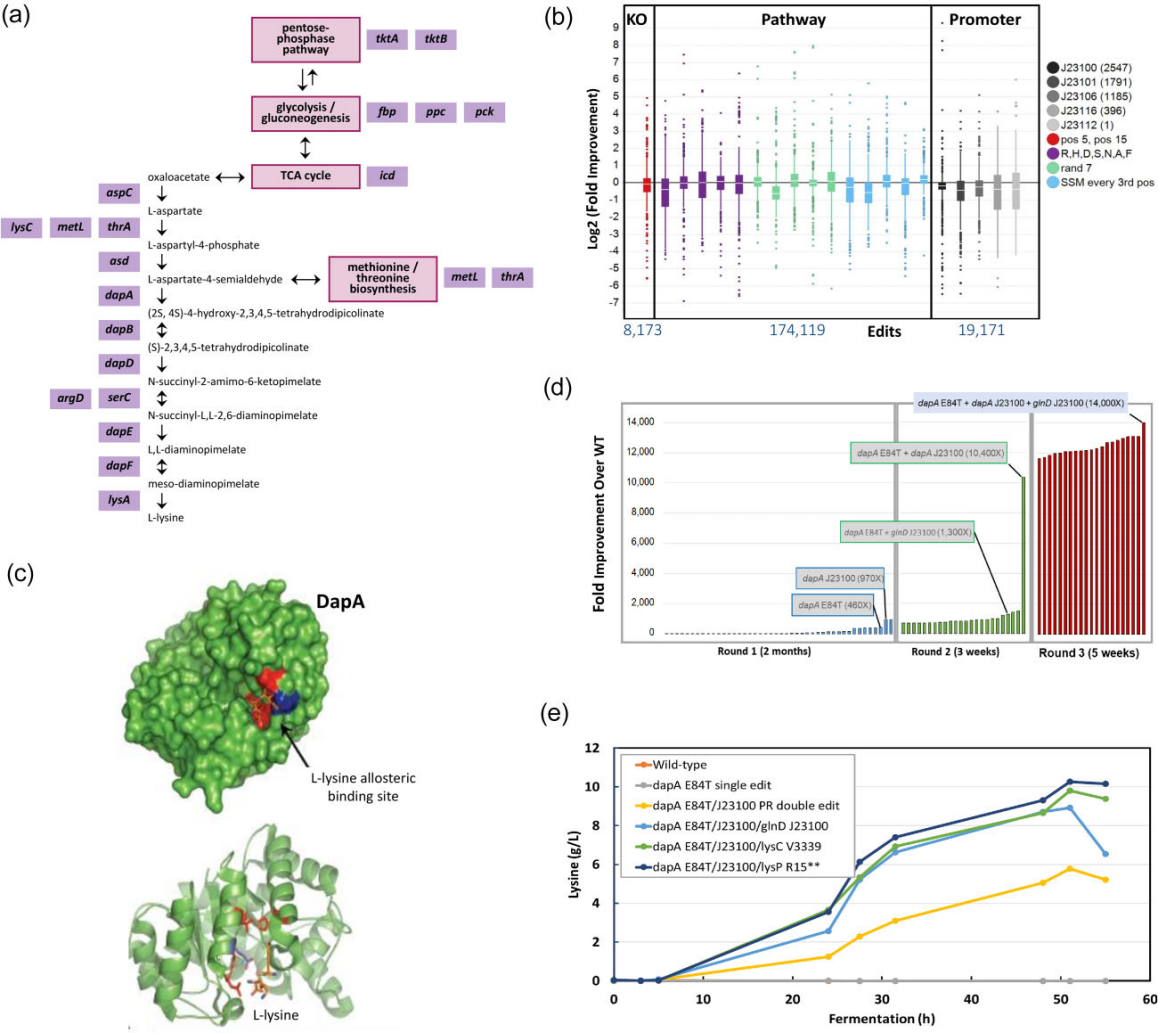
(a) The *E. coli* lysine biosynthesis pathway, including upstream pathways involved in carbon and nitrogen flow into the lysine pathway. (b) Screening of 18 000 variants from genome-wide lysine libraries comprising a total of 200 000 edits identified hits across all target types. (c) DapA (DHDPS) surface projection (left) and ribbon projection (right) show that the hits from the site-saturation libraries map to the lysine allosteric binding site, with multiple residues corresponding to site-specific hits highlighted in red and the E84 residue in blue. The orange structures represent lysine molecules bound to the allosteric binding site (Soares da Costa et al., 2016). (d) The top hits from the first, second, and third rounds of genome engineering show a stepwise improvement in lysine production. (e) The results of microplate screening were confirmed in 2-L batch fermentation experiments.

The genome-wide libraries used to engineer the strain for lysine overproduction introduced edits at the protein, pathway, and whole-genome levels (Fig. 7a). At the single-gene level, we focused on a well-known key regulatory node within the lysine biosynthetic pathway, *dapA*, encoding the dihydrodipicolinate synthase (DHDPS) enzyme. The site saturation mutagenesis library for *dapA* included 5802 designs to substitute every wild-type amino acid with each of the other 19 amino acids. On the pathway level, the genes in the lysine biosynthetic pathway, along with select central metabolism genes, were targeted via randomized site saturation libraries of three different types: (1) site-saturation mutagenesis of every third codon position; (2) codon replacement at every position to 7 randomly selected amino acids; and (3) codon replacement to 7 assigned amino acids (arginine (R), histidine (H), aspartic acid (D), serine (S), asparagine (N), alanine (A), and phenylalanine (F)) at every position. On the genome-wide level, all protein-coding genes in the *E. coli* genome were targeted for either knockout or altered expression using promoters of various strengths inserted in front of every CDS. A total of 24 different libraries were generated comprising approximately 200 000 unique and precise edits.

Following the introduction of edits into the base strain, 18 000 random isolates were screened in microtiter plants for increased lysine production using Agilent's RapidFire High-throughput Mass Spectrometry System (Fig. 7b). Strain isolates from each library were screened for improved lysine production after 24 hr of growth in minimal media, identifying several unique hits with up to 970-fold improvement in lysine titers that spanned all library types (including knockout, promoter insertion, and site-saturation mutagenesis libraries) and targets from single-gene to genome level. One of the best-performing variants identified after the first round of editing and screening was the *dapA* E84T variant from the saturation mutagenesis library (460-fold increase in lysine titer). The substitution of threonine for glutamate at position 84 has been previously reported in the literature to improve lysine production (Geng et al., 2013). This position, along with several other hits found in the *dapA* site-saturation library, can be mapped to a lysine allosteric inhibitory site of the DapA enzyme (Fig. 7c). Another top hit came from the promoter insertion library, where a strong constitutive J23100 synthetic promoter was inserted in front of the *dapA* gene (970-fold increase in titer). In both cases, the lysine titer improvement resulted from the editing of a single pathway enzyme by alleviating feedback negative inhibition of this key pathway node.

The *dapA* E84T variant was taken as a base strain for the second round of editing. The second-round libraries were designed based on hits identified in the first round. Deep screening of this library identified variants with up to 10 400-fold titer improvement compared to the wild type (Fig. 7d). The best-performing variant combined the two top hits identified in the first round of screening, *dapA* E84T and *dapA* J23100, further supporting the hypothesis that the regulatory function of DapA can be bypassed through overexpression or alleviation of allosteric inhibition. Finally, the third round of editing was done using the *dapA* E84T + *dapA* J23100 strain and libraries containing the top hits from the first round of editing. The screening of the edited strains revealed variants with further improved titers of up to 14 000-fold. The top three lysine producing variants were evaluated for growth and production in 2-L stirred tank fermentation cultivations and demonstrate that the titer improvements identified in the high-throughput screening format were maintained during scale-up, with the top strain reaching 10 g/L lysine titer (Fig. 7e).

### Strain Engineering for Small Molecule Production

Natural products are widely used in cosmetics, personal care products, and as APIs, constituting a multi-billion-dollar market (Transparency Market Research, 2021) with significant growth potential. Manufacturing these products in microbial strains can help create more sustainable, secure, and scalable supply chains to meet growing global demand. Strain engineering for small molecule production usually combines rational and exploratory approaches, which can include engineering specific enzymes, metabolic pathways, and regulatory networks, as well as complex phenotypes like tolerance and fitness. Successful examples of engineering natural product synthesis routes in yeast have included artemisinin (Ro, 2006), resveratrol (Li et al., 2015), noscapine (Li et al., 2018), breviscapine (Liu et al., 2018), opioids (Galanie et al., 2015; Pyne et al., 2020), and many others. As is the case for protein production, achieving commercially feasible strain performance requires a holistic approach incorporating both rational and whole genome approaches, in combination with the necessary phenotyping and predictive models to select manufacturing-robust strains.

**Fig. 8.**
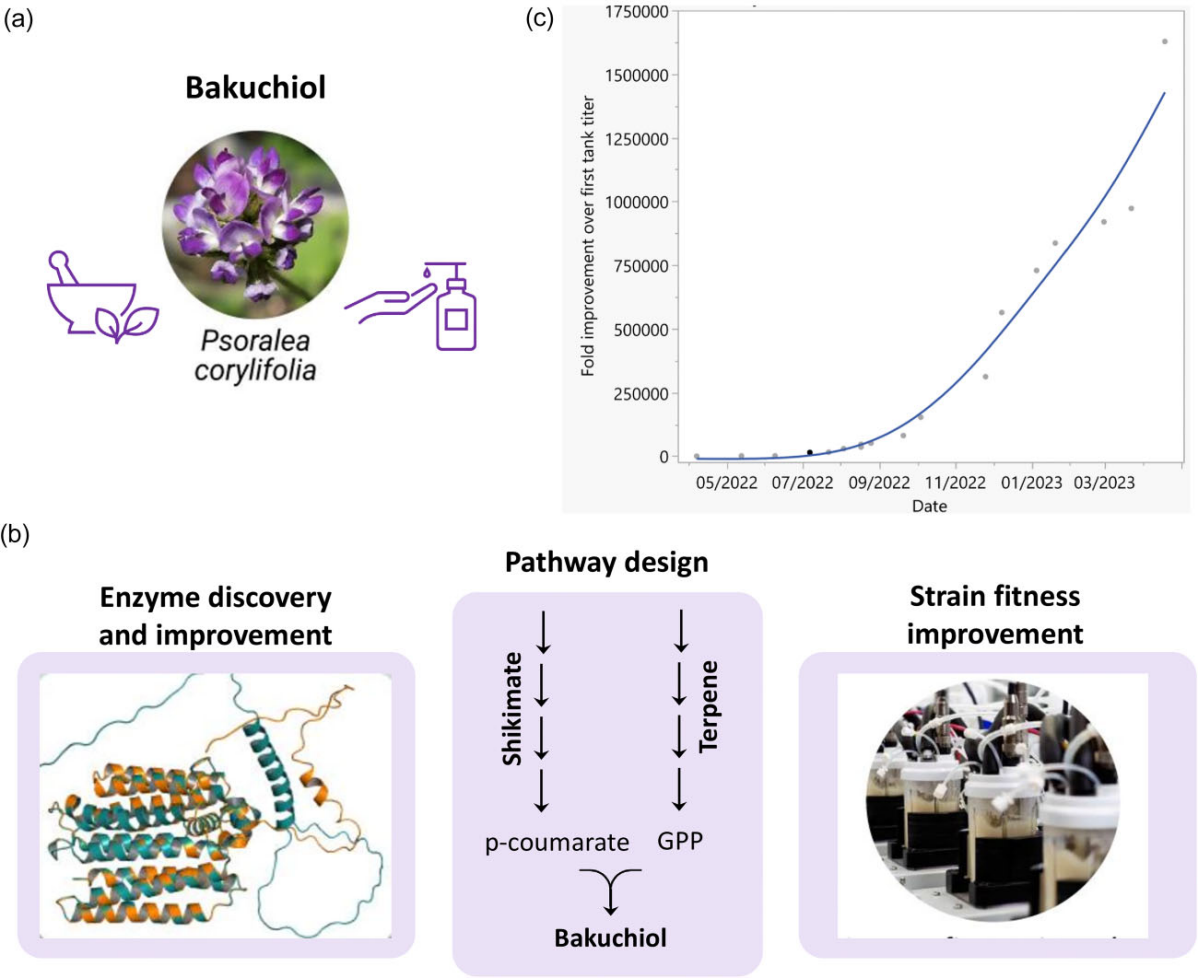
(a) Bakuchiol is a natural compound produced by the plant *Psoralea corylifolia* that has a significant potential for use in skincare applications. (b) Bakuchiol strain development steps included: (1) discovery and engineering of the key biosynthesis enzyme; (2) pathway design, including the identification and engineering of precursor routes; and (3) development of a robust *S. cerevisiae* production strain. (c) Strain engineering progress showing improvement in the bakuchiol production titer in screening bioreactors (250 mL) over a 12-month period.

To demonstrate the effectiveness of this approach in practice, we have chosen the small molecule bakuchiol as an example of engineering production of a plant-based natural product in *S. cerevisiae* (Adarsh Krishna et al., 2022). Bakuchiol is a meroterpene extracted from *Psoralea corylifolia* (Fig. 8a). It has attracted significant interest in the cosmetic industry due to its beneficial properties for skin care that are similar to those of retinol but without side effects, including UV sensitivity and irritation. The bakuchiol molecule was first identified in 1966 at the National Chemistry Laboratory in Pune, India, and named after the bakuchi plant (*P. corylifolia*), which has been used in ayurvedic and traditional Chinese medicine for centuries. Bakuchiol was introduced to the cosmetic markets in North America and Europe in 2007 and has been gaining popularity ever since. The growing demand for this ingredient has pushed the price up while putting pressure on the plant from which it is sourced. Microbial production of bakuchiol thus presents an attractive opportunity for achieving a renewable and scalable supply of the compound.

At the time the project was initiated, the pathway for the biosynthesis of this molecule was not fully elucidated and microbial production of bakuchiol had not been reported. In order to de-risk investment in other aspects of strain development, we initially focused our efforts on identifying the bakuchiol synthase that converts the two precursors—*p*-coumarate and geranyl diphosphate (GPP)—into bakuchiol (Fig. 8b). We identified a large panel of enzyme candidates through a bioinformatics process, triaged based on inclusion criteria and followed by high-sensitivity screening, which led to the discovery of the bakuchiol synthase. The initial microbially produced bakuchiol titer was barely detectable and the enzyme required significant activity improvement. This was done through *in vivo* enzyme saturation mutagenesis, which was carried out in the production strain itself using CREATE-based genome editing to identify a collection of single beneficial edits, followed by combinatorial library screening to identify synergistic effects. In addition to enzyme engineering of the synthase and other key enzymes, we employed rational, semi-rational, and genome-wide engineering approaches to improve and balance flux through the precursor pathways (the terpene and shikimate pathways)​, eliminate side products, and improve fitness. After multiple rounds of genome engineering and strain selection guided by advanced predictive models, we were able to obtain a scale-up-ready strain producing grams per liter of bakuchiol in less than 12 months (Fig. 8c).

## Conclusions

Advances in genome engineering have enabled strain development gains at much faster and greater scales than ever before. However, despite the increases in throughput, strain improvement is still largely an empirical process. Biology, unlike other disciplines, cannot be engineered in the true sense of the word, which is why our ability to predictably attain desired phenotypes and performance remains limited. While there may not be a universal set of biological design principles, we are beginning to understand how to make the DBTL process more effective and efficient. This is done by increasing both the quantity and quality of strains we can build and gathering high-quality, multidimensional data necessary to understand which design solutions contribute to the best outcomes. Looking at the entire DBTL cycle to eliminate bottlenecks and match the throughput at each stage, rather than optimizing individual steps, can significantly reduce the time and cost to scale-up and manufacturing.

One of the biggest remaining challenges in industrial strain engineering is scaling production from bench scale to commercial reactors without losing performance (National Research Council et al., 2015). The current industry benchmark for the strain development process estimates 5 years and approximately ${\$}$50 million to bring a product to market (Chang, 2021). A significant part of the resources and time are wasted on failed scale-up runs, in which strains are selected for scale-up based on the data obtained in small-scale experiments. This happens because growth in industrial bioreactor conditions may select for strains that avoid the fitness cost of diverting large amounts of carbon and energy to the product, resulting in genetic escape and loss of product biosynthetic capacity. Therefore, it is critical to develop methods for predicting strain performance at the bioreactor scale without risking thousands of dollars on failed bioreactor test runs, and each engineering cycle should evaluate not only the candidate strain's titer and yield but its fitness and scale-up potential as well (Chubukov et al., 2016).

At the turn of this century, Craig Venter coined the famous phrase: “If the 20th century was the century of physics, the 21st century will be the century of biology” (Venter & Cohen, 2004). What will it take to realize this vision? High-throughput genome exploration combined with effective screening and AI methods is helping improve our ability to reliably engineer biological systems. These systems are complex, yet with the advent of precise high-throughput data generation and machine learning tools, we can begin to predict their behavior without having to fully understand the underlying mechanisms. With the help of effective organism engineering approaches, we can begin to unlock the full potential of biomanufacturing to power the 21st century.
